# Orbitofrontal Signaling of Future Reward is Associated with Hyperactivity in Attention-Deficit/Hyperactivity Disorder

**DOI:** 10.1523/JNEUROSCI.0411-18.2018

**Published:** 2018-07-25

**Authors:** Jana Tegelbeckers, Martin Kanowski, Kerstin Krauel, John-Dylan Haynes, Carolin Breitling, Hans-Henning Flechtner, Thorsten Kahnt

**Affiliations:** ^1^Departments of Children and Adolescent Psychiatry,; ^2^Neurology, Otto von Guericke University Magdeburg, 39120 Magdeburg, Germany,; ^3^Center for Behavioral Brain Sciences, 39106 Magdeburg, Germany,; ^4^Bernstein Center for Computational Neuroscience,; ^5^Berlin Center for Advanced Neuroimaging, Charité–University Medical Center Berlin, 10115 Berlin, Germany,; ^6^Berlin School of Mind and Brain, Humboldt University of Berlin, 10117 Berlin, Germany,; ^7^Department of Neurology,; ^8^Department of Psychiatry and Behavioral Sciences, Feinberg School of Medicine, Northwestern University, Chicago, Illinois 60611, and; ^9^Department of Psychology, Weinberg College of Arts and Sciences, Northwestern University, Evanston, Illinois 60208

**Keywords:** ADHD, fMRI, goal-directed behavior, motivation, orbitofrontal cortex, reward

## Abstract

Alterations in motivated behavior are a hallmark of attention-deficit/hyperactivity disorder (ADHD), one of the most common psychiatric disorders in children and adolescents. The orbitofrontal cortex (OFC) plays a key role in controlling goal-directed behavior, but the link between OFC dysfunction and behavioral deficits in ADHD, particularly in adolescence, remains poorly understood. Here we used advanced high-resolution functional magnetic resonance imaging (fMRI) of the human OFC in adolescents with ADHD and typically developing (TD) controls (*N* = 39, age 12–16, all male except for one female per group) to study reward-related OFC responses and how they relate to behavioral dysfunction in ADHD. During fMRI data acquisition, participants performed a simple decision-making task, allowing us to image expectation-related responses to small and large monetary outcomes. Across all participants, we observed significant signal increases to large versus small expected rewards in the OFC. These responses were significantly enhanced in ADHD relative to TD participants. Moreover, stronger reward-related activity was correlated with individual differences in hyperactive/impulsive symptoms in the ADHD group, whereas high cognitive ability was associated with normalized OFC responses. These results provide evidence for the importance of OFC dysfunctions in the neuropathology of ADHD, highlighting the role of OFC-dependent goal-directed control mechanisms in this disorder.

**SIGNIFICANCE STATEMENT** Attention-deficit/hyperactivity disorder (ADHD) is characterized by alterations in motivated behavior which can be understood as diminished goal-directed control. The orbitofrontal cortex (OFC) plays a key role in controlling goal-directed behavior, but its potential contribution to ADHD symptomatology remains poorly understood. Using high-resolution fMRI, we show that adolescent ADHD patients display enhanced OFC signaling of future rewards and that these increased reward-related responses are correlated with the severity of hyperactivity/impulsivity. These findings suggest that an inability to adequately evaluate future outcomes may translate into maladaptive behavior in ADHD patients. They also challenge the idea that dysfunctions in dopaminergic brain areas are the sole contributor to reward-related symptoms in ADHD and point to a central contribution of goal-directed control circuits in hyperactivity.

## Introduction

Attention-deficit/hyperactivity disorder (ADHD) is one of the most common childhood psychiatric disorders (Polanczyk et al., 2007). In addition to prominent deficits in executive functioning, inhibition, attention, and motor regulation, dysfunctional motivational and reward processes are a hallmark of ADHD (Sagvolden et al., 2005; Luman et al., 2010). ADHD patients typically prefer small immediate rewards over large delayed rewards, make riskier choices to obtain reward, and are particularly responsive to positive reinforcement (for review, see Luman et al., 2010). Current models integrate these findings and suggest a link between ADHD symptom severity and altered reward processing (Sonuga-Barke, 2011; Plichta and Scheres, 2014).

These behavioral alterations have been interpreted as reflecting increased reward sensitivity and impulsivity, and, thus, previous work on the neuropathology of reward processing in ADHD has focused primarily on dopaminergic brain regions. For instance, reward anticipation in adolescents (Scheres et al., 2007; van Hulst et al., 2017) and adults (Ströhle et al., 2008; Plichta and Scheres, 2014) with ADHD has frequently been shown to correlate with decreased striatal activity, and effective pharmacological treatments for ADHD enhance dopamine availability by blocking dopamine reuptake (Krause et al., 2000; Volkow et al., 2001).

However, deficits in reward-related behavior in ADHD could also reflect deficient goal-directed control of behavior. The orbitofrontal cortex (OFC) is a key region supporting goal-directed behavior (Wallis, 2007; Rudebeck and Murray, 2014; Stalnaker et al., 2015). OFC neurons encode information about expected outcomes (Padoa-Schioppa and Assad, 2006; Stalnaker et al., 2014; Howard et al., 2015) and signal their current value depending on the state of the organism (Gottfried et al., 2003; Rudebeck and Murray, 2014; Howard and Kahnt, 2017). Accordingly, lesions to the OFC are associated with impulsive decision making and maladaptive behavior (Winstanley et al., 2004; Rudebeck et al., 2013). This raises the possibility that OFC dysfunction, resulting in an inability to adequately represent the value of future outcomes, might account for executive impairments in goal-directed behavior observed in ADHD.

However, evidence linking OFC activity to ADHD is sparse and inconsistent, particularly in adolescent populations. Some studies have found that OFC responses to monetary outcomes (Ströhle et al., 2008; Edel et al., 2013; von Rhein et al., 2015) and rewarding feedback (Dibbets et al., 2009; Cubillo et al., 2012) may be altered in ADHD, while others have found no differences (Stoy et al., 2011; Wilbertz et al., 2012). Moreover, most studies have not observed OFC activation in ADHD during expectation of monetary outcomes (Ströhle et al., 2008; von Rhein et al., 2015) or reported no differences compared with healthy control subjects (Stoy et al., 2011).

Inconsistent findings relating OFC function to ADHD could be due, in part, to technical difficulties in imaging the human OFC using functional magnetic resonance imaging (fMRI; Cortese et al., 2012). Due to its close proximity to nasal and paranasal sinuses, fMRI signals in the OFC are susceptible to signal dropout and geometric distortions (Deichmann et al., 2003; Weiskopf et al., 2006). However, recent advances in fMRI sequence development, including reduced field-of-view (rFOV) and parallel imaging (Heidemann et al., 2012), now allow more sensitive and reliable measurements of OFC activity.

In this study, we used advanced high-resolution rFOV fMRI of the OFC and a simple decision-making task (Kahnt et al., 2010) to test whether encoding of expected reward in the OFC is altered in adolescents with ADHD relative to typically developing (TD) adolescents. Moreover, we tested whether potential differences in OFC activity are associated with relevant diagnostic parameters, such as inattentive and/or hyperactive symptoms (Edel et al., 2013), aggressive/dissocial behaviors (Rubia et al., 2009), age, and cognitive ability (Stoy et al., 2011). Finally, we performed voxel-based morphometry (VBM) to test whether structural abnormalities resulting from a potential prefrontal maturational delay (Shaw et al., 2007) might explain altered OFC activity in ADHD.

## Materials and Methods

### 

#### 

##### Participants.

Forty-nine children and adolescents (27 ADHD, 22 TD) aged between 12 and 16 years participated in the study. They were recruited through local newspaper advertisements and the Department of Child and Adolescent Psychiatry and Psychotherapy at Otto von Guericke University Magdeburg. All participants and their parents underwent clinical interviews with the Revised Schedule for Affective Disorders and Schizophrenia for School-Age Children–Present and Lifetime Version (K-SADS-PL; Kaufmann et al., 1997) for assessment of psychiatric disorders based on DSM criteria. Attentional deficits were further evaluated using the Youth Self-Report (YSR; self-rating; Achenbach, 1991a) and the Child Behavior Checklist (CBCL; parental rating; Achenbach, 1991b). Performance in standardized measures of intelligence [Culture Fair Intelligence Test–Revised Version (CFT-20R); Weiss, 2008] and attentional capacity (d2; Brickenkamp, 2002) was assessed in all participants. Subjects with an intelligence quotient (IQ) <85 or >130 (3x ADHD, 2x TD [three ADHD and two TD participants were excluded based on this criterion]), any current or previous neurological or psychiatric disorder other than ADHD or oppositional defiant disorder or conduct disorder (CD) accompanying ADHD, substance abuse, or severe head motion during fMRI scanning (2x ADHD [two ADHD participants were excluded based on this criterion]) were excluded. In total, seven participants were excluded based on these criteria. Moreover, three ADHD patients were excluded based on task performance (<4 correct trials per condition in >3 runs). The final ADHD sample (*N* = 19) consisted of 10 patients who fulfilled the diagnostic criteria for the combined subtype of ADHD, 7 patients who fulfilled criteria for the inattentive subtype, and 2 patients of the hyperactive-impulsive subtype. All subjects in the final sample were male, except for one female participant in each group.

Based on the information about parental employment from the CBCL questionnaire, the estimated household income of ADHD patients was lower compared to TD families (*t*_(37)_ = −2.67, *p* = 0.011). Although these estimates do not provide insight into the immediate finances of the adolescents (e.g., allowance), they can serve as a proxy for the financial situation of the family and might therefore correlate with the subjective value of the monetary outcomes used in the experiment. Parental ratings of aggressive and dissocial behaviors were significantly higher in the ADHD group than the TD group (Table 1), but only two patients fulfilled the criteria for oppositional defiant disorder. ADHD patients currently medicated with stimulants (*N* = 3, lifetime *N* = 7) discontinued the intake at least 48 h before the experiment. Table 1 describes the final study sample.

**Table 1.** Sample characteristics

	ADHD	TD	*t* (*p*)
*N*	19 (1 female)	20 (1 female)	
Age	14.11	14.6	−1.19 (0.24)
IQ (CFT-20R)	102.95	110.45	−2.22 (0.03)
Attentional capacity (d2, *t* value)	52.58	62.70	−3.82 (0.00)
Attentional problems			
Self-rating (YSR; *t* value)	61.67	53.25	4.44 (0.00)
Parental rating (CBCL; *t* value)	69.18	52.50	7.68 (0.00)
Aggression			
Self-rating (YSR; *t* value)	55.61	52.25	1.77 (0.09)
Parental rating (CBCL; *t* value)	61.65	52.7	4.14 (0.00)
Dissocial behavior			
Self-rating (YSR; *t* value)	54.78	52.15	1.49 (0.15)
Parental rating (CBCL; *t* value)	56.12	52.5	2.35 (0.02)

The study was approved by the local ethics committee of the medical faculty at Otto von Guericke University Magdeburg. All participants and parents gave written informed assent/consent. Participants received a voucher for a local shopping mall as reimbursement (€5 per hour) in addition to their earnings from the experimental task.

##### Experimental task design.

**Figure 1.**
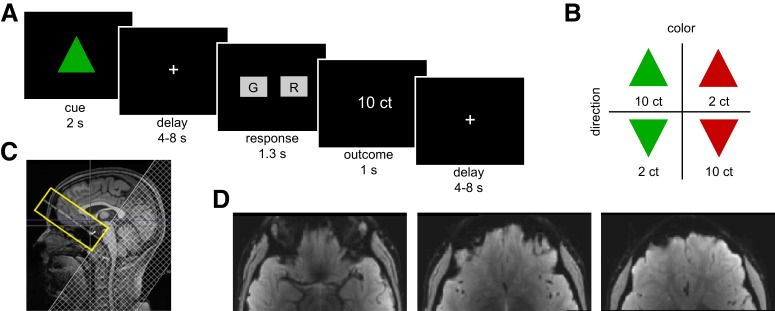
Experimental design and the optimized rFOV imaging protocol. ***A***, Illustration of experimental task structure. ***B***, Examples of the visual dimensions of the CSs predicting reward. For half of the participants, the depicted CS-value association was inverted. ***C***, Midsagittal view of a sample subject showing the rFOV (yellow) over the OFC and the saturation band (hatched area) for outer volume suppression with a sharp transition edge at the side slightly overlapping with the rFOV. ***D***, Mean functional rFOV echoplanar image of a representative subject. ct, Cents.

Participants completed a decision-making task in which four different triangle shapes served as visual conditioned stimuli (CSs; green or red triangles that pointed either upward or downward; Fig. 1*B*). These CSs were associated with large and small monetary rewards (10¢ and 2¢, respectively). CSs were counterbalanced across subjects such that for half of the participants, red upward and green downward triangles were associated with large rewards, and red downward and green upward triangles were associated with small rewards. On each trial, one CS was presented for 2 s, and participants were instructed to remember the color and orientation of the CS for a variable delay (4–8 s). This delay allowed us to dissociate CS- and outcome-evoked activity. Participants then had to report either the color or orientation of the CS by button press with the right index or middle finger within an interval of 1.5 s to gain the monetary reward predicted by the CS. The chosen response was briefly surrounded by a white frame to indicate the participant's selection, and feedback about the monetary outcome was presented if the response was correct (Fig. 1*A*). If the response was incorrect or too slow, feedback of 0¢ was presented.

To gain familiarity with the task and to learn the associations between the CSs and monetary outcomes, all participants performed a training session before MRI scanning (consisting of 4 runs with 12 trials each). Subsequently, subjects performed 7 runs with 24 trials each (six presentations of each triangle in pseudorandomized order) while fMRI data were acquired. At the end of each experimental run, the total gain was displayed. The entire experimental session, including the instructions, training, anatomical scan, and behavioral task, lasted ∼1.5 h.

##### MRI data acquisition.

MRI data were collected on a Siemens Prisma 3 Tesla system with a 64-channel head/neck coil. An Original Pillow Junior (Tempur World) was placed on the base of the coil surrounding the sides and the back of the head for comfort and to prevent movement.

T1-weighted structural images were acquired with an MPRAGE sequence using the following parameters: 1 × 1 × 1 mm^3^ voxel size, 256 × 256 × 192 matrix, 2.82 ms echo time, 2.5 s repetition time (TR), 1.1 s inversion time, 7° flip angle, 140 Hz pixel bandwidth, 7/8 partial Fourier, and parallel imaging with a Generalized Autocalibrating Partial Parallel Acquisition (GRAPPA) factor of 3.

For high-resolution functional imaging, we used a combination (Heidemann et al., 2012) of rFOV gradient EPI and parallel imaging to minimize signal dropout, geometric distortion, and loss of blood oxygen level–dependent (BOLD) contrast in the OFC. This imaging sequence is part of the Siemens “Advanced fMRI” work-in-progress software. During each of the seven functional runs, we acquired 190 volumes using the following parameters: 24 slices without interslice gap, 1.8 mm slice thickness, interleaved slice acquisition order, 120 × 240 mm^2^ rFOV, 1.25 × 1.25 mm^2^ in-plane voxel size, 30 ms echo time, 2 s TR, 90° flip angle, 1 mT/m · ms *z* shim, 0.73 ms echo spacing, and GRAPPA factor of 2. The transversal slice block was tilted 20° with respect to the anterior commissure–posterior commissure line as shown in Figure 1*C*. An example of a mean functional image for one participant is shown in Figure 1*D*.

To facilitate anatomical registration of the rFOV imaging data, we also acquired 10 whole-brain EPI volumes. To keep geometrical distortions within the rFOV part of the whole brain data set identical to the functional scans, the shim settings were copied and the FOV and matrix size in phase direction as well as the GRAPPA factor were doubled.

##### MRI data preprocessing.

All analyses of the MRI data were conducted using SPM12 (RRID: SCR_007037, Wellcome Trust Centre for Neuroimaging, London, UK). For preprocessing, all functional rFOV echoplanar images were realigned to the first volume. Independent sample *t* tests on mean absolute head motion revealed group differences in translation (*t*_(37)_ = 4.36, *p* < 0.001) and rotation (*t*_(37)_ = 4.27, *p* < 0.001). To control for potential signal differences induced by head motion, motion parameters were included in first- and second-level statistical models (see section *Statistical analysis of functional MRI data* below).

We used the whole-brain echoplanar images to facilitate spatial normalization of the rFOV EPIs. Specifically, the 10 whole-brain echoplanar images were motion corrected, averaged, and coregistered to the T1 structural image. Next, the functional rFOV EPI time series was coregistered to the mean whole-brain echoplanar image using the mean rFOV echoplanar image. Spatial normalization was then performed by normalizing the T1-weighted structural image to MNI (Montreal Neurological Institute) space using the six tissue probability map provided by SPM12. Deformation fields were applied to the functional rFOV echoplanar images. The normalized images were smoothed with a 6 mm FWHM Gaussian kernel.

##### Statistical analysis of functional MRI data.

For each participant, a general linear model (GLM) was fitted to the normalized and smoothed functional rFOV EPI data. The GLM included the following six event-related regressors: the onsets of the visual CSs (separately for large and small rewards), button presses, and outcomes [separately for large, small, and no rewards (incorrect responses)]. These regressors were convolved with a canonical hemodynamic response function. The GLM also included the following nuisance regressors to account for head motion: six realignment parameters, their squares, their derivatives, and their squared derivatives (total 24). In addition, within-volume motion was estimated using the absolute difference between odd and even slices, as well as the variance across slices, and included in the GLM. Within-volume motion estimates were also used to identify volumes with excessive motion (more than four times SD), which were modeled separately using additional volume-specific regressors.

Voxelwise parameter estimates from this GLM reflect the amplitude of the BOLD signal in response to events of interest (CSs and outcomes). The BOLD signal is related to the overall level of neural activity evoked by these events. Individual contrast images for CSs predicting large versus small rewards (expectation) and large versus small outcomes (outcome) were computed based on these parameter estimates. The outcome contrast included correct responses only as incorrect trials were modeled in a separate regressor (no reward). Contrasts were entered into one-sample *t* tests across all participants for group analysis. To account for differences in head motion, we included absolute head motion as a covariate of no interest.

We used an inclusive mask of the OFC, defined as the conjunction of an anatomical OFC mask (Anatomical Automatic Labeling atlas) and a gray matter (GM) mask (SPM's tissue probability map, thresholded at >0.20). Significant clusters were defined using a statistical threshold of *p* < 0.05, familywise error (FWE) corrected at the voxel-level. Individual parameter estimates were extracted from the OFC (cluster defined across all subjects at *p* < 0.001, uncorrected) and compared between groups using an ANOVA with group (ADHD vs TD) and reward value (high vs low) as factors.

To relate OFC activity to IQ and ADHD symptomatology, we used a stepwise multiple linear regression analysis. The initial regression model included age, IQ, number of hyperactive/impulsive and inattentive symptoms (K-SADS-PL rating of the parental interviews), and *T* values for dissocial behavior and aggression (CBCL). For variables included in the final regression model, Pearson's correlations coefficients between the parameter estimates and these variables were computed.

##### Statistical analysis of structural MRI data.

We tested for group differences in GM volume using VBM (Ashburner and Friston, 2005). The preprocessing followed the manual of the CAT12 toolbox (http://www.neuro.uni-jena.de/cat/): each image was automatically normalized into template space and segmented into GM, white matter, and CSF. The individual whole-brain volume for each tissue type and total intracranial volume (TIV) was extracted. After a quality check of the individual images and a check for homogeneity between the groups, the images were smoothed with an 8 mm FWHM Gaussian kernel. Finally, the groups were compared using independent-sample *t* tests, with TIV as a covariate to correct for overall differences in brain volume. Furthermore, we performed automatic segmentation of regions of interest based on Hammer's atlas as provided by CAT12. GM volume in left and right OFC was compared between groups (independent-sample *t* test) and related to age (Pearson's correlation).

## Results

### Behavioral results

**Figure 2.**
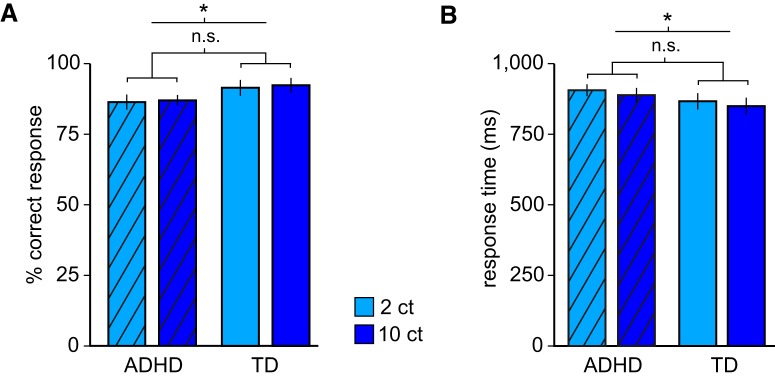
Behavioral results. ***A***, Mean accuracy for trials with small and large rewards in ADHD and TD participants, showing a significant main effect of group but no main effect of reward value and no group-by-value interaction. ***B***, Mean response times for small and large rewards in ADHD and TD participants, showing that ADHD patients responded significantly slower than TD participants, and that larger rewards were associated with faster response times in both groups. There was no significant group-by-value interaction. *p < 0.05. n.s., nonsignificant; ct, cents. Error bars indicate SEM.

Participants reported the correct color or orientation with high accuracy, indicating that they attended to the task (average, 89.33% correct; one-sample *t* test against 50% chance, *t*_(38)_ = 46.54, *p* < 0.001). A two-way ANOVA with the factors *reward value* (2¢ vs 10¢) and *group* (ADHD vs TD) on the percentage of correct responses revealed an overall inferior performance of the ADHD group compared to TD participants (*F*_(1,37)_ = 12.4, *p* = 0.001), but no effect of reward value (*F*_(1,37)_ = 0.58, *p* = 0.45) and no group-by-value interaction (*F*_(1,37)_ = 0.02, *p* = 0.89; Fig. 2*A*). Analysis of response times (RTs) showed that ADHD patients responded slower than TD participants (*F*_(1,37)_ = 4.13, *p* = 0.049). However, RTs in both groups were shorter in the high-value than in the low-value condition (*F*_(1,37)_ = 5.08, *p* = 0.03), with no group-by-value interaction (*F*_(1,37)_ = 0.02, *p* = 0.89), demonstrating that CS-induced reward expectations had comparable effects on behavioral responses in both groups (Fig. 2*B*). Overall, these results suggest that even though ADHD patients showed unspecific behavioral deficits (possibly related to deficits in attention or working memory) relative to TD participants, all participants were able to perform the task, and that the value of expected rewards had comparable behavioral effects on both groups. Importantly, the absence of group-by-value interactions indicates that subsequent analyses of the imaging data are not confounded by behavioral group differences.

### Increased OFC responses to expected rewards in ADHD

**Figure 3.**
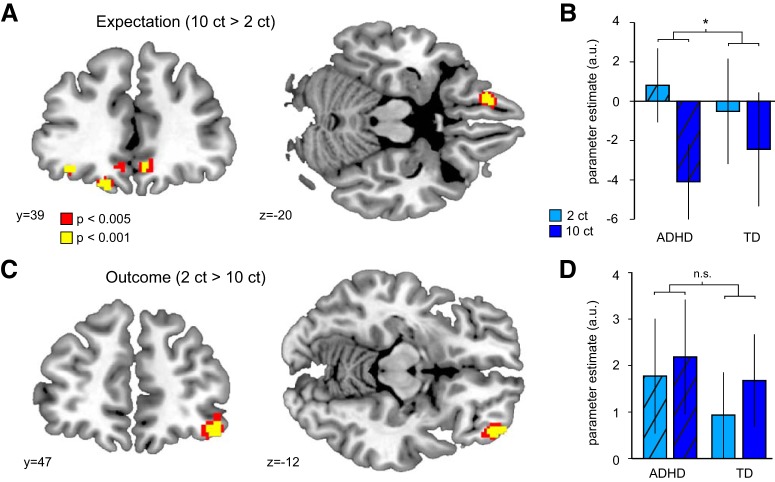
fMRI responses to reward expectation and outcome. ***A***, Responses in OFC to large versus small expected rewards in adolescents with ADHD and TD peers. ***B***, Parameter estimates [arbitrary units (a.u.)] for responses to large versus small expected rewards in the left OFC differed between ADHD and TD groups (significant group-by-value interaction). ***C***, Responses in the OFC to feedback of small versus large monetary outcomes in ADHD and TD participants. ***D***, Parameter estimates for responses to large versus small reward outcomes in the OFC did not differ between ADHD and TD groups. *p < 0.05. n.s., nonsignificant; ct, cents. Error bars indicate SEM.

In a first step, we tested whether OFC responses differed between CSs predicting large versus small outcomes across the entire group of participants. This analysis revealed a significant cluster in the left OFC (BA11, *x* = −16, *y* = 42, *z* = −20; *t*_(37)_ = 5.79, *p*_FWE_ = 0.004; Fig. 3*A*). Additional bilateral OFC clusters did not survive correction for multiple comparisons (Table 2 shows results at *p* < 0.001, uncorrected). We did not find regions in the OFC that were more responsive to small compared to large expected rewards.

**Table 2.** OFC responses to expectation of large versus small outcomes across all participants (*p* < 0.001, *k* > 10)

	*k*	MNI (in mm)			*t*	*p*_FWE_
Left OFC	40	−16	42	−20	5.79	0.004
Left OFC	13	−36	38	−14	4.25	0.168
Right OFC	14	24	48	−10	4.61	0.074
Right OFC	18	6	38	−12	4.19	0.190

We next tested whether OFC responses to expected rewards differed between groups. A two-way ANOVA on parameter estimates in the left OFC cluster defined above revealed a significant interaction between *reward value* and *group* (*F*_(1,37)_ = 5.76, *p* = 0.022). As illustrated in Figure 3*B*, OFC responses to large versus small expected rewards were stronger in the ADHD group, and there was no significant main effect of *group* (*F*_(1,37)_ = 0.01, *p* = 0.92). We confirmed this finding using non-parametric statistical tests [Wilcoxon rank-sum, *p* = 0.0197; permutation (*N* = 10,000), *p* = 0.0117]. Note that this analysis is unbiased, such that the OFC cluster in which responses were compared between groups was defined independently of group differences (Kriegeskorte et al., 2009). Moreover, a model with IQ as a covariate revealed that group differences in expectation-related activity in the OFC were influenced by IQ but survived as a trend (*t*_(36)_ = 1.68, *p* = 0.10, two-tailed). However, ADHD and lower cognitive ability, as measured by IQ tests, are genetically codetermined (Kuntsi et al., 2004), and statistically controlling for IQ when comparing ADHD with TD peers is therefore not generally advisable (Dennis et al., 2009), as this would remove variance that is intrinsically related to the disorder. A weakening of the observed effect was therefore expected. Finally, the effect remained significant when controlling for household income (*t*_(36)_ = 2.22, *p* = 0.033), suggesting that potential differences in the subjective value of the monetary rewards did not account for our results (Tobler et al., 2007). These results demonstrate that compared to TD adolescents, adolescents with ADHD show increased OFC responses to stimuli predicting large versus small rewards and that this effect is not fully explained by IQ or participants' financial background.

### No group differences in OFC responses to reward outcomes

In a second step, we examined OFC responses to reward feedback. Across all subjects, we found significantly increased activity in the right lateral OFC (BA11, *x* = 44, *y* = 48, *z* = −14; *t*_(37)_ = 5.23, *p*_FWE_ = 0.017; Fig. 3*C*) in response to small compared to large outcomes.

However, a two-way ANOVA on parameter estimates extracted from this OFC cluster revealed no significant differences between groups (main effect of *group*, *F*_(1,37)_ = 0.77, *p* = 0.39; group by outcome value *interaction*, *F*_(1,37)_ = 2.33, *p* = 0.14). We did not find regions in the OFC that were more responsive to large compared to small reward outcomes.

### Reward-related responses in OFC are related to ADHD symptoms

Having established altered OFC responses in ADHD participants compared to TD participants, we next tested whether responses in the ADHD group were associated with relevant diagnostic parameters (IQ, age, hyperactivity/impulsivity, inattentiveness, aggressive or dissocial behavior).

**Figure 4.**
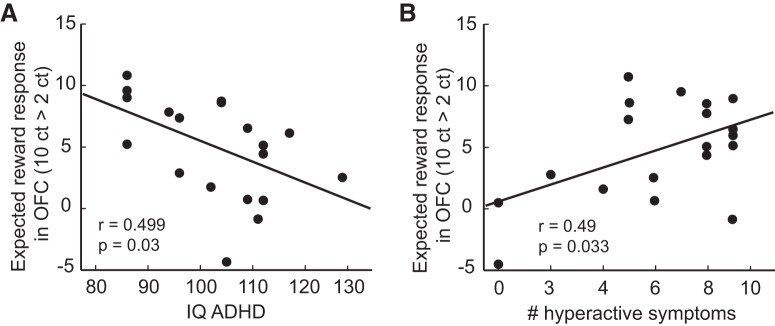
Reward-related fMRI responses in OFC predict diagnostic characteristics in ADHD. ***A***, OFC responses to expected reward (10¢ vs 2¢) correlate with intellectual ability in adolescents with ADHD. ***B***, OFC responses to expected reward (10¢ vs 2¢) correlate with hyperactivity based on parental rating in the ADHD group. ct, Cents.

Using stepwise linear regression, we found that fMRI responses to large versus small expected rewards were best explained (*R*^2^ = 0.573, *F*_(2,14)_ = 9.375, *p* = 0.003) by a combination of IQ (β = −0.423, *p* = 0.032) and the number of hyperactive/impulsive symptoms (β = 0.558, *p* = 0.007). None of the other variables including age, inattentiveness, and aggressive and dissocial behaviors significantly increased the model fit. Subsequent correlation analyses confirmed a negative correlation between the expectation-related OFC responses and IQ (*r* = −0.499, *p* = 0.03; Fig. 4*A*) and a positive correlation with the number of hyperactive/impulsive symptoms (*r* = 0.49, *p* = 0.033; Fig. 4*B*) in the ADHD group. These findings suggest that hyperactivity/impulsivity is related to alterations in OFC reward processing, whereas cognitive capacity is associated with reduced OFC activity during reward expectation.

In contrast to CS-related responses, a stepwise linear regression on OFC responses to large versus small reward outcomes revealed no significant effects of any of the predictors.

### Influence of gray matter volume on reward processing

In a final step, we examined whether group differences in reward-related responses could be explained by differences in GM volume. Whole-brain and left OFC GM volume were negatively correlated with age across all participants (whole-brain, *r* = −0.367, *p* = 0.021; left OFC, *r* = −0.38, *p* = 0.017; right OFC, *r* = −0.291, *p* = 0.072). However, there were no significant group differences between TD and ADHD adolescents in either whole-brain or OFC GM volumes (*p* values >0.34). This suggests that ongoing structural brain organization affects ADHD and TD adolescents to a comparable degree. In addition, controlling for age, OFC responses to large versus small expected rewards did not correlate with GM volume in the OFC (left OFC, *r* = 0.233, *p* = 0.16; right OFC, *r* = 0.106, *p* = 0.53) or the whole brain (*r* = 0.121, *p* = 0.47).

## Discussion

By signaling the current value of available options, the OFC plays an essential role in goal-directed behavior and decision making (Wallis, 2007; Stalnaker et al., 2015). However, its contribution to maladaptive and impulsive behavior in ADHD has remained unclear. Here we showed that OFC signaling of future rewards differs in adolescents with ADHD and TD age-matched peers. Specifically, we observed stronger responses to large versus small expected rewards in ADHD patients, suggesting enhanced signaling of expected value in this disorder.

Our findings suggest a potential role of enhanced reward signaling in ADHD pathology. OFC activity was correlated with ADHD symptoms, such that patients with particularly strong OFC responses to large versus small rewards displayed higher levels of hyperactivity/impulsivity. It is tempting to speculate that OFC activity during reward expectation could translate into potentially maladaptive behaviors by aberrant assignment of value to expected outcomes or goals. In theory, ascribing high value to goals could increase the likelihood of approach behavior in children with ADHD, such that patients who strongly represent the value of future rewards might not only display heightened reward sensitivity but may also experience waiting for rewards as particularly disturbing (delay aversion) and react with stimulation seeking as compensation (Sonuga-Barke, 2005). Future studies that are designed to directly examine impulsive behavior are needed to further explore this potential mechanism.

Imaging of the OFC is challenging because this area is highly susceptible to signal drop-out and geometric distortions (Deichmann et al., 2003; Weiskopf et al., 2006). These difficulties may explain why previous models of reward sensitivity in ADHD have focused primarily on the role of dopaminergic dysfunction and alterations in the striatum in driving behavioral deficits (for review, see Luman et al., 2010). These models are based on findings of diminished dopamine availability (Spencer et al., 2005; Fusar-Poli et al., 2012) and attenuated striatal responses to expected rewards in ADHD (Scheres et al., 2007; Ströhle et al., 2008; van Hulst et al., 2017). However, reduced responses in dopaminergic brain regions are difficult to reconcile with the fact that children with ADHD typically display enhanced behavioral responses to reward (Plichta and Scheres, 2014). Our data, acquired using an advanced fMRI sequence, point toward aberrant value signaling in the OFC as a potential mechanism underlying hyperactivity/impulsivity in ADHD patients. Diminished striatal responses combined with enhanced OFC signaling may create a disadvantageous imbalance between neural circuits controlling reward-related behaviors. Indeed, previous studies indicate that altered functional connectivity between the ventral striatum and the OFC may account for changes in reward-related behavior in ADHD (Tomasi and Volkow, 2012; Costa Dias et al., 2013).

Consistent with some (Stoy et al., 2011; Wilbertz et al., 2012) but not all previous reports in ADHD (Ströhle et al., 2008; von Rhein et al., 2015), we did not find changes in OFC responses to reward feedback. It is possible that inconsistencies among previous findings are related to difficulties in imaging the OFC in humans. It is therefore important to note that our rFOV fMRI sequence allowed us to measure OFC responses with almost no spatial distortions. Our findings add to this discussion by supporting the view that responses to reward outcomes are unaltered in ADHD (Scheres et al., 2007, Tripp and Wickens, 2009).

In addition, CS-evoked OFC responses were not related to aggressive or antisocial behaviors. Given that none of our patients fulfilled the diagnostic criteria for CD, this null result might be due to insufficient variance in our sample. However, it could also indicate that impulsivity associated with ADHD may rely on altered OFC encoding of value, whereas impulsivity underlying CD could rely on overall decreased OFC activity (Rubia et al., 2009). In line with this, ADHD patients are not impulsive per se, but rather make impulsive choices when this strategy reduces experienced delays (Sonuga-Barke et al., 1992).

Our results suggest a normalizing effect of intellectual ability on aberrant OFC responses in ADHD. This adds to previous studies reporting that differences in OFC activity between patients and TD peers were no longer evident when controlling for IQ (Rubia et al., 2009; Stoy et al., 2011). However, IQ values, as a product of multiple influences, should be considered as indices of global functioning (Dennis et al., 2009), and the correlation between OFC encoding of value and IQ suggests that specific neural processes may relate to global functioning in ADHD. Alternatively, working memory could be the common modulating factor between IQ and OFC encoding, as IQ and working memory are highly correlated constructs (Colom et al., 2004; Ackerman et al., 2005), and OFC has been linked to working memory for reward information (Miller and Cohen, 2001; Wallis, 2007).

Importantly, our results cannot be explained by differences in prefrontal development. VBM analyses confirmed that GM volume was comparable between ADHD and TD participants, ruling out an effect of potential delays in brain maturation in this ADHD sample (Shaw et al., 2007).

In conclusion, our study provides insights into how the OFC may contribute to altered reward-related behavior in adolescent ADHD. We show that enhanced OFC encoding of reward is associated with symptoms of hyperactivity/impulsivity. Our findings thus challenge the idea that dysfunctions in dopaminergic brain areas are the sole contributor to reward-related behavioral symptoms and suggest that aberrant activity in goal-directed control circuits may lie at the core of hyperactive/impulsive symptoms in ADHD.
